# Downregulation of MYCN through PI3K Inhibition in Mouse Models of Pediatric Neural Cancer

**DOI:** 10.3389/fonc.2015.00111

**Published:** 2015-05-12

**Authors:** Tene Aneka Cage, Yvan Chanthery, Louis Chesler, Matthew Grimmer, Zachary Knight, Kevan Shokat, William A. Weiss, W. Clay Gustafson

**Affiliations:** ^1^Department of Neurological Surgery, Brain Tumor Research Center, University of California San Francisco, San Francisco, CA, USA; ^2^Department of Neurology, University of California San Francisco, San Francisco, CA, USA; ^3^Department of Bioengineering, Santa Clara University, Santa Clara, CA, USA; ^4^Division of Paediatric Solid Tumour Biology and Therapeutics, The Institute of Cancer Research, London, UK; ^5^Department of Biochemistry, University of Southern California, Los Angeles, CA, USA; ^6^Department of Physiology, University of California San Francisco, San Francisco, CA, USA; ^7^Department of Cellular and Molecular Pharmacology, University of California San Francisco, San Francisco, CA, USA; ^8^Department of Pediatrics, University of California San Francisco, San Francisco, CA, USA; ^9^Department of Pediatric Hematology and Oncology, University of California San Francisco, San Francisco, CA, USA; ^10^Hellen Diller Family Comprehensive Cancer Center, University of California San Francisco, San Francisco, CA, USA

**Keywords:** pediatric, cancer, neuroblastoma, medulloblastoma, MYCN, PI3 kinase, cell signaling, mTOR

## Abstract

The *MYCN* proto-oncogene is associated with poor outcome across a broad range of pediatric tumors. While amplification of *MYCN* drives subsets of high-risk neuroblastoma and medulloblastoma, dysregulation of MYCN in medulloblastoma (in the absence of amplification) also contributes to pathogenesis. Since PI3K stabilizes MYCN, we have used inhibitors of PI3K to drive degradation. In this study, we show PI3K inhibitors by themselves induce cell cycle arrest, with modest induction of apoptosis. In screening inhibitors of PI3K against MYCN, we identified PIK-75 and its derivative, PW-12, inhibitors of both PI3K and of protein kinases, to be highly effective in destabilizing MYCN. To determine the effects of PW-12 treatment *in vivo*, we analyzed a genetically engineered mouse model for MYCN-driven neuroblastoma and a model of MYCN-driven medulloblastoma. PW-12 showed significant activity in both models, inducing vascular collapse and regression of medulloblastoma with prominent apoptosis in both models. These results demonstrate that inhibitors of lipid and protein kinases can drive apoptosis in MYCN-driven cancers and support the importance of MYCN as a therapeutic target.

## Introduction

The MYC family of proto-oncogenes (*MYC*, *MYCN*, and *MYCL*) has been broadly implicated in cancer. MYC proteins are helix–loop–helix transcription factors, which share a large degree of homology and function. MYC transcriptionally regulates ~500 core targets with as many as 2000 additional cancer and/or cell type-specific targets, making pharmacologic targeting downstream of MYC proteins challenging. However, MYC proteins are tightly regulated at the level of protein stability through several upstream signaling events (1). The *MYCN* gene is amplified or overexpressed in a number of pediatric cancers including both medulloblastoma and neuroblastoma and is a negative prognostic factor in both diseases (2, 3).

Neuroblastoma is the most common extracranial solid tumor of childhood. High-risk neuroblastoma represents about 40% of all patients diagnosed. Despite significant progress in the treatment of low- and intermediate-risk patients, survival among children with high-risk disease remains poor even after significant escalations in the intensity of therapy (4, 5). Amplification of *MYCN* occurs in ~25% of patients and is the clearest genetic risk factor for high-risk disease, making MYCN a prominent candidate for targeted therapies (6, 7). Targeted expression of *MYCN* to the peripheral neural crest of mice results in neuroblastoma tumors with high penetrance. We have previously shown that indirect methods of blocking MYCN in these animals have led to decreased tumor size and improved survival (8, 9).

Medulloblastoma is the most common malignant brain tumor in children and is divided into four molecular subgroups. *MYC* family oncogenes are amplified in ~10% of medulloblastoma tumors and amplification correlates with poor survival (10–12). *MYCN* is highly expressed in two of the medulloblastoma subgroups, the Sonic Hedgehog (SHH) group and the Group 4 tumors (13). Using a genetically engineered model (GEM), we have previously targeted expression of *MYCN* to the cerebellum of transgenic mice. These GTML mice are predisposed to SHH-independent, MYCN-amplified medulloblastoma (14). We have shown that transient downregulation of *MYCN* in these mice resulted in tumor cell senescence and improved survival (14, 15). In addition, medulloblastoma tumor cell lines with constitutive activation of the SHH pathway derived from Ptc1^+/−^p53^−/−^ mice (SmoWT) (16–18), demonstrate robust tumorigenesis (17). Loss of the Ptch receptor in these mice leads to constitutive activation of the downstream smoothened transmembrane protein, resulting in medulloblastomas driven by MYCN and GLI. This model provides an excellent platform to explore MYCN-directed pre-clinical therapeutics in neuroblastoma and in medulloblastoma.

As transcription factors with no surfaces amenable to small molecule binding, MYC proteins are widely considered undruggable directly. However, the stability of MYCN protein is tightly regulated by a sequential series of key phosphorylation events downstream of CDK1/CyclinB and the PI3K/mTOR pathway, suggesting inhibitors of relevant kinases as an approach to target MYC and MYCN (1). Further, these phosphorylation events are known to be downstream of the activating ALKF1174L mutation, which plays a role in both sporadic and familial neuroblastomas (19). We have previously shown that the tool PI3K inhibitor LY294002 and the dual PI3K/mTOR inhibitor NVP-BEZ235 cause downregulation of MYCN protein and, in the case of NVP-BEZ235, decreased angiogenesis through a paracrine signaling mechanism (9, 20). These studies emphasize the importance of mouse models for MYCN-driven pediatric cancers and implicate further exploration of the interplay between PI3K and MYCN protein regulation through the use of alternate pharmacologic inhibitors of PI3K.

Several specific inhibitors of PI3K have been developed and characterized in other systems including PI-103, PIK-75, PW-12, and BEZ235 (21, 22). Resistance to PI3K inhibitors is scaffold-dependent and often mediated by point mutations (23). If PI3K inhibition is to be a viable strategy in cancer, demonstration of efficacy across an array of PI3K inhibitor scaffolds will likely be necessary. We have previously shown efficacy for the PI3K/mTOR inhibitor BEZ235 in neuroblastoma (9). In the current study, we show that the PI3K alpha inhibitor PIK-75 and its derivative, PW-12, destabilize MYCN protein and induce cell cycle arrest and apoptosis in *MYCN*-amplified cell lines. We further demonstrate that these drugs have pre-therapeutic efficacy in models of *MYCN*-driven neuroblastoma (24) and *MYCN*-driven medulloblastoma (16, 25). These studies provide evidence for PI3K inhibition with subsequent blockade of MYCN and vascular collapse as a therapeutic strategy across chemical scaffolds and an impetus for the continued development of PI3K inhibitors in *MYCN*-driven pediatric cancers, including both neuroblastoma and medulloblastoma.

## Materials and Methods

### Immunoblotting

Human Kelly neuroblastoma cell lines were obtained from the University of California at San Francisco. Cells were grown in RPMI medium with 10% FBS at 37°C. In most experiments, cells were conditioned in 2% FBS for 5 h and replaced with full medium and recombinant human insulin-like growth factor-I (20 ng/ml) (Invitrogen, Waltham, MA, USA) for 1 h before harvesting. Lysates were collected and sonicated. Primary antibodies were as follows: anti-MYCN, anti-phosphorylated Akt, anti-p-rpS6, anti-Akt, anti-rpS6 (Cell Signaling Technology, Danvers, MA, USA), and β-tubulin (Upstate, Buffalo, NY, USA). Immunoblots were developed with horseradish peroxidase-conjugated secondary antibodies (Calbiochem, San Diego, CA, USA) and Enhanced Chemiluminescence Plus reagents (Amersham, Piscataway, NJ, USA).

### Cell proliferation assay and flow cytometry

Kelly neuroblastoma cells were suspended in basal RPMI medium with 10% FBS and plated in 96-well tissue culture plates (Corning/Sigma-Aldrich, Tewksbury, MA, USA). To measure viable cells, Kelly cells were incubated at 37°C for 16 h and then changed into fresh medium containing LY-294002 (Sigma, St. Louis, MO, USA), PI-103 (Calbiochem, San Diego, CA, USA), PIK-75, PW-12 (Kevan Shokat, Ph.D. Lab, University of California San Francisco, CA, USA), or DMSO vehicle. We analyzed DNA content at 48 h after plating using Water Soluable Tetrazolium (WST-1) Cell Proliferation Assay Kit (Invitrogen, Grand Island, NY, USA) according to the manufacturer’s protocol. In this assay, A_450 nm_ − A_800 nm_ is proportional to secreted WST-1 activity and proliferation. In addition, cell cycle effects were measured using flow cytometry as previously described in our lab (26). For these experiments, Kelly neuroblastoma cells were treated for 48 h with 1 μM of PI-103 or PW-12.

### Neuroblastoma genetically engineered mouse model

*TH-MYCN* transgenic mice were maintained in hemizygotic matings as previously described (24). All animals were housed and treated following University of California at San Francisco Institute on Animal Care and Use Committee (IACUC) guidelines. TH-MYCN mice with neuroblastoma tumors were treated either with 25 mg/kg PW-12 in 10% DMSO/water (*n* = 3) or vehicle alone [10% DMSO/water (*n* = 3)], once daily by IP injection, for 14 days. For Western blot analysis, tumors were snap-frozen at the time of harvesting. For immunohistochemistry, tumors were fixed in 10% formalin for 24 h and processed as described below. For tumor weight assessment, tumors were weighed on the final day of treatment, 14 days. Tumor diameters were measured on day 14 and volumes were calculated (mm^3^ = width^2^ × length/2).

### Shh medulloblastoma subgroup tumor model

All athymic nude mice were obtained from Jackson Laboratories (Bar Harbor, ME, USA), and were housed and treated following University of California at San Francisco Institute on Animal Care and Use Committee (IACUC) guidelines. About 6 × 10^6^ SmoWT medulloblastoma cells derived from medulloblastoma tumors generated from Ptc1^+/−^p53^+/−^ mice (16–18) were injected subcutaneously into the flank of athymic nude mice. A total of 10 mice were used for these experiments. Starting 8 h after implantation of cells, five animals were treated with 25 mg/kg PW-12 in 10% DMSO/water and five were treated with vehicle alone [(10% DMSO/water)], once daily by IP injection for 7 days. Tumor diameters were measured with calipers daily and volumes were calculated (mm^3^ = width^2^ × length/2).

### Histology

For H&E analysis, 10-μm sections were cut paraffin blocks. Hematoxylin (Vector Labs, Burlingame, CA, USA) and bluing agent was added. Slides were washed three times with water and once with 70% ethanol (EtOH) for 5 min each. Eosin was added followed by 70, 95, and 100% EtOH, and dipped into xylene. Slides were mounted with Vectashield (Vector Labs) and analyzed with light microscopy. For immunohistochemistry, samples were prepared as described previously (20) using the following antibodies: Ki67 (1:200; M7249; Dako, Carpinteria, CA, USA), Ki67 (1:100; NB600-1252; Novus Biologicals, Littleton, CO, USA), cleaved-Caspase-3 (1:200; 9661; Cell Signaling Technology, Danvers, MA, USA), MYCN (1:50; 10159-2-AP; Proteintech, Chicago, IL, USA), and CD-31 (1:50; 55024; BD Bioschences, San Diego, CA, USA).

### Statistical analysis

To evaluate *in vivo* data, averages were calculated and error bars were generated using SD. Student’s *t*-test was used to calculate statistical significance. A *p*-value <0.05 was considered statistically significant.

## Results

### Both PIK-75 and PW-12 potently downregulate MYCN protein

In order to assess the effects of pharmacologic inhibition of PI3K on *MYCN*-driven cells, human Kelly *MYCN*-amplified neuroblastoma cells were treated with various PI3K inhibitors: PI-103, LY-294002, PIK-75, and PW-12. PI-103 causes proliferative arrest but not apoptosis during *in vivo* glioma treatment (26). It acts by blocking class I PI3Ks and mTOR, and subsequent downstream stabilization of MYCN protein. LY-294002 also causes proliferative arrest but not apoptosis. It is also a pan-selective, broad-spectrum PI3K/mTOR inhibitor that blocks both p-Akt and the downstream target of mTOR p-rpS6 (22). LY-294002 is associated with significant cellular toxicity and therefore has limited effect as viable human clinical *in vivo* agent (26, 27). However, in xenograft models of neuroblastoma and glioma, it has been shown to effectively block progression of tumorigenesis though not apoptosis (26, 28, 29). PIK-75 is a PI3 kinase alpha selective inhibitor, specifically, it is an imidazopyridine inhibitor that induces apoptosis, not growth arrest, in glioma cells (29). However, this inhibitor carries significant cellular toxicity with broad off-target effects and therefore is not a viable clinical therapeutic option. Because of this, PIK-75 is an effective agent to study *in vitro*, but is toxic *in vivo*. PW-12, a derivative of PIK-75, induces growth arrest as well as some apoptosis. It has fewer off-target effects than its counterpart and therefore is a viable option for *in vivo* animal and human clinical therapeutics.

**Figure 1:**
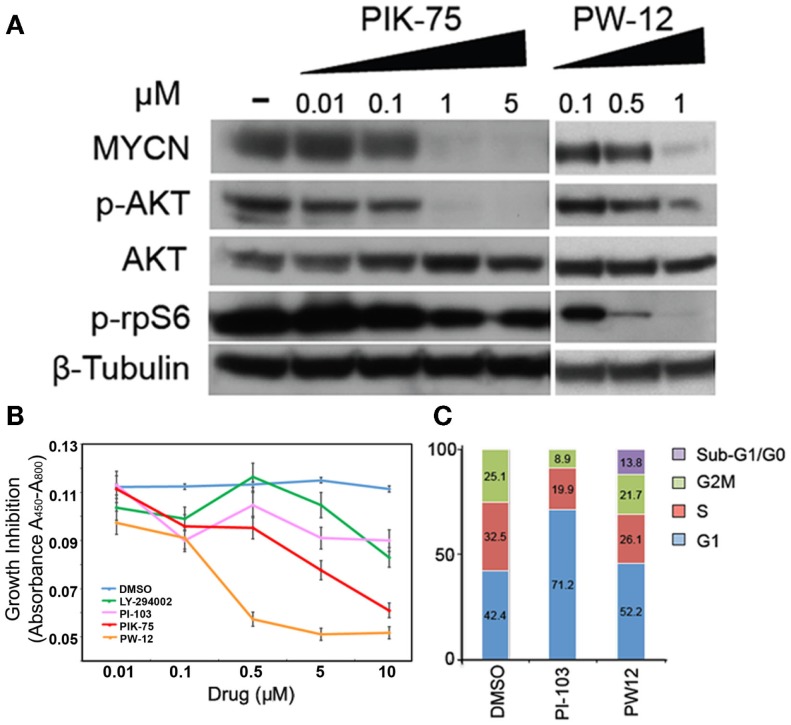
**Inhibitors of PI3K downregulate MYCN protein, increase apoptosis, and decrease viability in MYCN-amplified tumor cells**. **(A)** Kelly MYCN-amplified neuroblastoma cells were treated for 24 h with the indicated concentrations of inhibitor (PIK-75 or PW-12) and blotted for MYCN and markers downstream of PI3K (pAKT) and mTOR (prpS6). **(B)** Kelly neuroblastoma cells were treated with inhibitor (LY-294002, PI-103, PIK-75, PW-12, or DMSO) for 48 h and cell proliferation was measured by WST1 assay. **(C)** Kelly neuroblastoma cells were treated with 1 μM of inhibitor (PI-103, PW-12) for 48 h and cell cycle effects were analyzed by flow cytometry.

Human Kelly *MYCN*-amplified neuroblastoma cells were treated with increasing concentrations of PIK-75 or PW-12 and blotted for markers of PI3K activity and MYCN protein (Figure 1A). These two drugs were chosen from the PI3K panel because PIK-75 is a highly effective *in vitro* inhibitor but due to known toxicity is not a viable *in vivo* option. PW-12, however, is a less toxic derivative of PIK-75. There is clear downregulation of MYCN in response to both PIK-75 and PW-12 (Figure 1A). This corresponds directly with concomitant downregulation of AKT phosphorylation downstream of PI3K. These data indicate that the stability of MYCN protein is sensitive to inhibition of PI3K alpha *in vitro* as well as some mTOR inhibition as evidenced by blockade of p-rpS6. Further, blockade of PI3K by PIK-75 results in a significant decrease in viability in *MYCN*-amplified cells (Figure 1B) when PW-12 and PIK-75 are compared to LY294002 (a tool compound PI3K/mTOR inhibitor with lower affinity for these kinases) or PI-103, a PI3K/mTOR inhibitor with intermediate activity.

Next, we explored two PI3K inhibitors to evaluate effects on cell cycle, PI-103 and the PIK-75 derivative PW-12. These were chosen because their low-toxicity profiles make them more viable therapeutic options than their counterparts. When compared to the control, PW-12 demonstrated significant apoptosis, as measured by flow cytometry and measurement of the sub-G1/G0 fraction (Figure 1C), suggesting increased apoptosis as one contributor to the increase in activity when comparing PW-12 to PI-103 and to vehicle. This loss of cells to apoptosis could explain the differences in cell cycle profiles between PI-103 and PW-12. Notably, MYCN is critical for S-phase entry in MYCN-driven disease and both PW-12 and PIK-75 result in a loss of cells in S-phase and accumulation of cells in G1, perhaps because of increased potency against PI3K by PW-12 in these cells (Figure 1A). In addition, the smaller percentage of Kelly neuroblastoma cells treated with PW-12 are in the S and M phases of the cell cycle suggesting decreased proliferation.

### PI3K inhibition by PW-12 has efficacy in a TH-MYCN model of neuroblastoma

**Figure 2:**
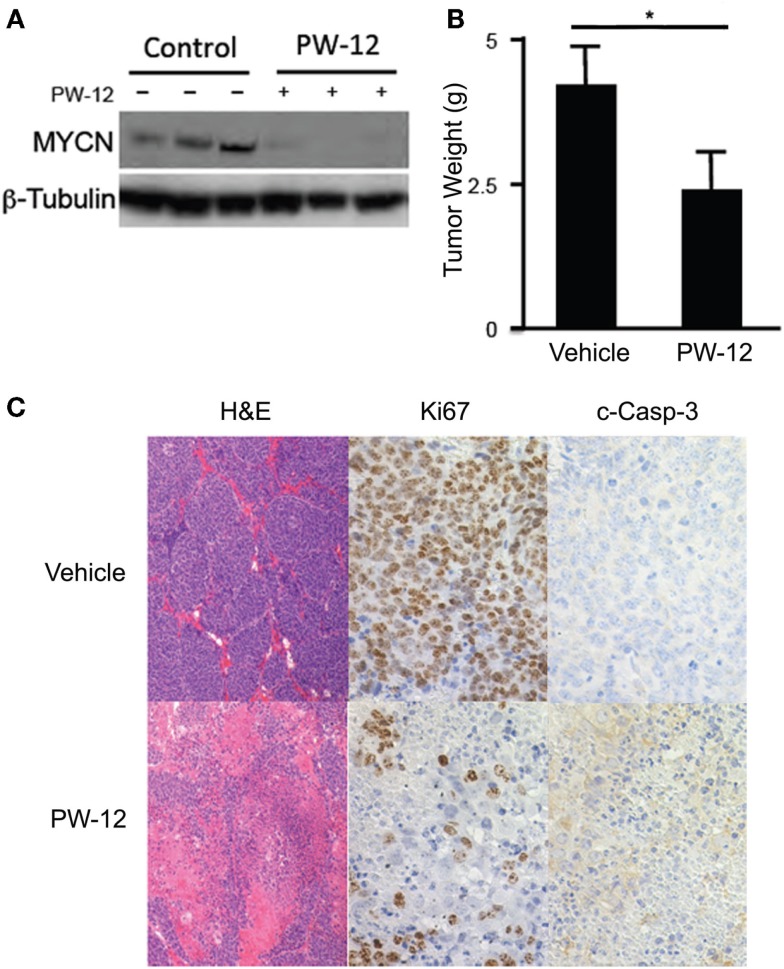
**PW-12 is efficacious in a GEM model of MYCN-amplified neuroblastoma**. Tumor-bearing TH-MYCN mice were treated for 14 days with either 25 mg/kg of PW-12 (*n* = 3) or vehicle alone (*n* = 3). **(A)** Western blot for MYCN protein from treated tumors confirm that PW-12 is effective at downregulating MYCN protein production. **(B)** Tumor size was significantly reduced in treated animals (* represents *p* < 0.05). **(C)** Histology for Ki67 and c-Caspase 3 shows increase in apoptosis and decrease in proliferation in treated tumors.

Using the TH-MYCN neuroblastoma mouse model in which human *MYCN* is expressed under control of the mouse tyrosine hydroxylase promoter, we are able to generate mouse neuroblastoma tumors with high penetrance, which are genetically, histologically, and morphologically similar to human neuroblastomas (24). Abdominal tumors were detected in TH-MYCN mice by palpation and either PW-12 (25 mg/kg) or vehicle alone was administered by IP injection daily for 14 days. PW-12 treated tumors demonstrate a significant decrease in MYCN protein expression and in tumor mass (*p* < 0.05) when compared to vehicle treated controls (Figures 2A,B), consistent with direct blockade of MYCN and subsequent anti-tumor effects. They also showed decreased cellularity, decreased proliferation measured by Ki-67 staining, and increased apoptosis as measured by cleaved caspase 3 staining (Figure 2C). These results support the role of PW-12 in neuroblastoma tumor regression, thereby positioning it to be a pre-clinical therapeutic for MYCN-driven neuroblastoma.

### PI3K inhibition by PW-12 has efficacy in a MYCN-driven SHH medulloblastoma

**Figure 3:**
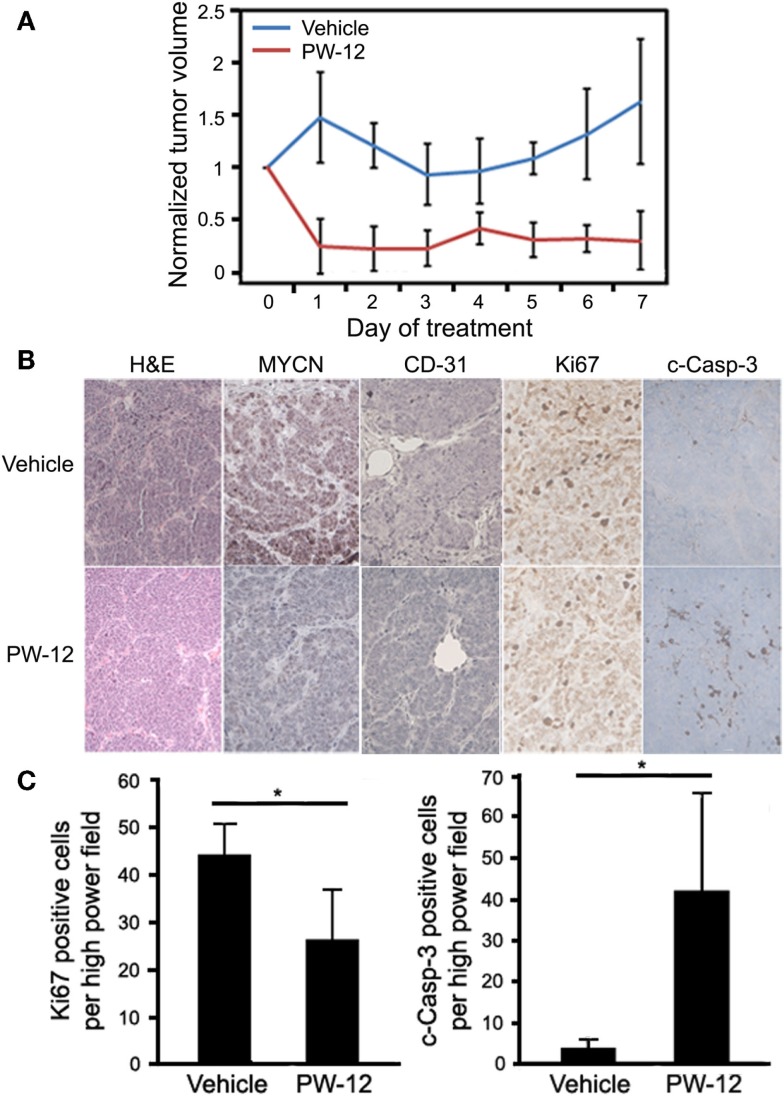
**PW-12 is efficacious in an orthotopic flank tumor model of MYCN-amplified medulloblastoma**. Mice were implanted with 6 × 10^6^ SmoWT medulloblastoma cells in the hindflank. Animals were treated for 7 days with 25 mg/kg of PW-12 (*n* = 5) or vehicle alone (*n* = 5). **(A)** Tumor size was significantly reduced in treated animals (all time points days 1–7 resulted in statistically significant differences between the treatment and control groups). **(B)** Histology on H&E stain is consistent with medulloblastoma (H&E, 20×). Tumor cell proliferation (Ki67, 40×) was decreased, apoptosis (cleaved-caspase-3, 40×) increased, MYCN (20×) expression downregulated, and vascularity decreased in PW-12 treated tumors as confirmed by histology **(B)** and cell quantification for apoptosis and cell proliferation **(C)**. (* represents*p* < 0.05).

To study MYCN-driven medulloblastoma, we used a subcutaneous flank allograft model where SmoWT cells were injected in to the flank of athymic nude mice. In this model, SmoWT cells from tumors of Ptc1^+/−^p53^+/−^ mice undergo tumorigenesis via activation of the Shh pathway. Allografted animals were treated with either PW-12 (25 mg/kg) or vehicle by IP injection daily for 7 days. In response to therapy, treated tumors showed a statistically significant (*p* < 0.05) decrease in tumor volume (Figure 3A) with preserved medulloblastoma histology features on H&E (Figure 3B). The small amount of tumor mass in the treated tumors precluded us from immunoblotting these specimens. However, immunohistochemistry revealed uniform and profoundly decreased levels of MYCN across tumors in all five mice treated with PW-12 as well as decreased vascularity in treated tumors as measured by CD-31 compared to untreated tumors (Figure 3B). In addition, treated tumors showed a clear decrease in cell proliferation by Ki67 and marked induction of apoptosis, cleaved caspase (Figures 3B,C). These results establish the flank SmoWT tumor cell implantation as a useful model for pre-clinical therapeutics in medulloblastoma and suggest a potential for inhibitors of PI3K in children with *MYCN*-driven disease.

## Discussion

Effective pharmacologic targeting of the MYC family of transcription factor oncogenes, particularly targeting MYCN in pediatric cancer, has been a long sought after goal (1). Pre-clinical testing of drugs across pharmacologic scaffolds is an indicator of robustness of a particular drug target and mechanism as well as a potential means of bypassing emergent resistance. PI3K inhibition has previously been implicated in stabilization of MYCN. Here, we show that PW-12, a bioavailable derivative of the PI3K inhibitor PIK-75, shows knockdown of MYCN in a neuroblastoma cell line with marked reduction in viability and changes in cell cycle consistent with a MYCN-specific effect. Using a transgenic model of *MYCN*-amplified neuroblastoma and a model of medulloblastoma where murine-derived allografted cells are implanted into mice, we demonstrate significant *in vivo* efficacy against both neuroblastoma and medulloblastoma tumors. We further show on-target efficacy against tumor MYCN protein, marked decrease in proliferation and increase in tumor apoptosis as well as apparent tumor vascular collapse.

The past decade of experience with targeted inhibitors has shown that emergent resistance is nearly inevitable. Effective blockade of a particular pharmacologic target requires an array of pharmacologic scaffolds to bypass resistance by point mutation within the target, a resistance mechanism that has been shown to be active for PI3K inhibitors (23). To our knowledge, this is the first *in vivo* pre-therapeutic trial of PW12 in neuroblastoma or medulloblastoma and the first implicating blockade of MYCN in its effect. By broadening *in vivo* and cell line studies across this new pharmacologic scaffold, these studies add to the growing evidence implicating PI3K/mTOR inhibition as a viable strategy in MYCN-driven pediatric cancers including both neuroblastoma and medulloblastoma.

## Conflict of Interest Statement

The authors declare that the research was conducted in the absence of any commercial or financial relationships that could be construed as a potential conflict of interest.
